# Immune-related signature of periodontitis and Alzheimer’s disease linkage

**DOI:** 10.3389/fgene.2023.1230245

**Published:** 2023-10-02

**Authors:** Jieqi Jin, Mengkai Guang, Simin Li, Yong Liu, Liwei Zhang, Bo Zhang, Menglin Cheng, Gerhard Schmalz, Xiaofeng Huang

**Affiliations:** ^1^ Department of Stomatology, Beijing Friendship Hospital, Capital Medical University, Beijing, China; ^2^ Department of Stomatology, China-Japan Friendship Hospital, Beijing, China; ^3^ Stomatological Hospital, Southern Medical University, Guangzhou, China; ^4^ Department of Cariology, Endodontology and Periodontology, Leipzig University, Leipzig, Germany

**Keywords:** Alzheimer’s disease, periodontitis, neuroinflammation, multimorbidity, immune pathways, gene expression signature

## Abstract

**Background:** Periodontits (PD) and Alzheimer’s disease (AD) are both associated with ageing and clinical studies increasingly evidence their association. However, specific mechanisms underlying this association remain undeciphered, and immune-related processes are purported to play a signifcant role. The accrual of publicly available transcriptomic datasets permits secondary analysis and the application of data-mining and bioinformatic tools for biological discovery.

**Aim:** The present study aimed to leverage publicly available transcriptomic datasets and databases, and apply a series of bioinformatic analysis to identify a robust signature of immune-related signature of PD and AD linkage.

**Methods:** We downloaded gene-expresssion data pertaining PD and AD and identified crosstalk genes. We constructed a protein-protein network analysis, applied immune cell enrichment analysis, and predicted crosstalk immune-related genes and infiltrating immune cells. Next, we applied consisent cluster analysis and performed immune cell bias analysis, followed by LASSO regression to select biomarker immune-related genes.

**Results:** The results showed a 3 gene set comprising of DUSP14, F13A1 and SELE as a robust immune-related signature. Macrophages M2 and NKT, B-cells, CD4^+^ memory T-cells and CD8^+^ naive T-cells emerged as key immune cells linking PD with AD.

**Conclusion:** Candidate immune-related biomarker genes and immune cells central to the assocation of PD with AD were identified, and merit investigation in experimental and clinical research.

## Introduction

With the rapid ageing of global populations, the burden of Alzheimer’s disease (AD) is rising. AD is a neurodegenerative disease marked by the formation of amyloid-β peptide (AβP) plaques aggregate in brain tissues. Inflammation and pathological aberrations in central and peripheral immune responses are implicated in AD (Campbell and Gear, 1995; Bettcher et al., 2021). While the relationship of systemic or peripheral inflammation with AD has been inconsistent (Eriksson et al., 2011), accruing research has highlighted the role of peripheral inflammation in AD pathogenesis. Gut microbiome dysbiosis is associated with neuroinflammation and synaptic dysfunction characteristic of AD (Bairamian et al., 2022). Research using a murine model of AD has demonstrated that low-grade peripheral inflammation is capable of aggravating brain pathology (Xie et al., 2021). ApoE4 allele of the Apolipoprotein E gene, a well-known genetic risk factor of AD, when coupled with chronic low-grade peripheral inflammation leads to earlier onset and greater morbidity from AD (Tao et al., 2018). Peripheral inflammation also alters the connectivity of large-scale cognitive networks in older individuals, particularly in ApoE4 carriers (Walker et al., 2020). Elevated levels of both peripheral and CSF inflammatory markers are associated with AD (Shen et al., 2019). Systemic infections are associated with enhanced immunosuppressive processes in the brains of patients with AD, with an increase in anti-inflammatory proteins including IL4R and CHI3L1 and a decrease in certain proinflammatory proteins, along with lowered T-cell recruitment (Rakic et al., 2018). Systemic inflammation can affect intra-brain drug distribution by altering ABCB1 and ABCG2 protein expression and can also perturb GluN1 protein expression in AD affected brains (Puris et al., 2021). Circulating IL-21, a key immunomodulatory cytokine is elevated in AD, possibly due to immune activation, resulting in neuroinflammation, microglial activation, and deposition of Aβ plaques (Agrawal et al., 2022).

Ageing is associated with higher levels of chronic inflammation and immune deregulation. Infections cause immune dysregulation, an increase circulating pro-inflammatory mediators such as TNFα and IL-6 along with the brain levels of IL-1β and IL-6 levels, aggravating neuroinflammation and accelerating cognitive decline in older adults (Holmes et al., 2011; Lopez-Rodriguez et al., 2021). Immune perturbation in AD is not restricted to the central nervous system (CNS), and peripheral immune dysregulation appears to affect homeostasis in AD-affected brains, where the barrier function is disrupted, allowing an ingress of T-cells (van Olst et al., 2022). Perturbed naive and memory CD4^+^ T cell subsets have been noted in the peripheral blood of patients with mild AD and dementia, with a lower proportion of naive cells and an increased proportion of effector memory and terminal differentiation effector memory (TEMRA) CD4^+^ cells (McManus et al., 2015). The deregulation of both the peripheral and central immune compartments marks AD. Peripheral immune activation is associated with neuroinflammation and AD pathogenesis. Sustained activation of the brain’s microglia and other immune cells is found to exacerbate both amyloid and tau pathology and may serve as the link between infections, chronic peripheral inflammation and AD (Kinney et al., 2018).

Periodontitis is a highly prevalent oral infectious disease that imposes both oral and systemic health burdens. It is an inflammatory disease caused by a complex interplay between dental plaque microbes and the host immune system (Hajishengallis, 2014a). The deposition of a microbial plaque biofilm initiates immune cell migration, and its dysbiosis sustains a local inflammatory response (Murakami et al., 2018). Key periodontal pathogens such as Porphyromonas gingivalis are immune evasive and can activate the complement system and pathogen recognition receptors such as TLRs, leading to chronic inflammation and periodontal tissue destruction (Xu et al., 2021). Virulence factors like Porphyromonas gingivalis fimbriae can activate TLR2 expressed by innate immune cells (Maekawa et al., 2014), leading to a cascade of cellular and humoral immune responses, and induction of adaptive immune responses (Hajishengallis, 2014a). Ageing is associated with a steep increase in the incidence and severity of periodontitis, attributed in part to increased susceptibility from age-dependent alterations in host innate immunity and inflammatory status (Hajishengallis, 2014b). Cellular senescence, stem cell failure, and immune senescence inherent to biological ageing impair periodontal tissue homeostasis and contribute to the pathophysiology of periodontitis (Baima et al., 2022).

Evidence showing the association of AD with periodontitis is rapidly accumulating (Dominy et al., 2019; Dioguardi et al., 2020; Hu et al., 2021). Porphyromonas gingivalis has been found to infiltrate the brains of tissue in AD and is proposed to be an important mechanistic link between periodontitis and AD (Ryder, 2020). Periodontitis also causes widespread systemic immune dysfunction, showing heightened pro-inflammatory responses to Porphyromonas gingivalis and attenuated T-cell responses (Gaudilliere et al., 2019). In the present study, we aimed to identify immunological perturbations and immune crosstalk potentially linking periodontitis with AD by leveraging gene expression data.

## Materials and methods

### Gene expression datasets

We downloaded gene expression profile datasets related to periodontitis (PD) and Alzheimer’s disease (AD) from the GEO (https://www.ncbi.nlm.nih.gov/geo/) database. For PD, we chose gingival tissue and for AD, we chose brain tissue data. The datasets are listed in Table1.

**TABLE 1:** Statistical analysis of periodontitis and Alzheimer’s disease samples.

Disease	Datasets	Platform	Case	Control	Total
PD	GSE10334	GPL570	241	69	310
	GSE16134	GPL570	183	64	247
	GSE106090	GPL21827	6	6	12
AD	GSE33000	GPL4372	310	157	467
	GSE36980	GPL6244	33	47	80
	GSE122063	GPL16699	56	44	100
	GSE48350	GPL570	80	173	253
	GSE5281	GPL570	87	74	161

### Differential gene expression analysis

First, we converted the probe names into gene names based on the downloaded data. If the same gene had multiple expression values in the same sample, we obtained the mean of the expression values. As differences existed between the datasets, we first combined the datasets for AD and PD each based on common genes, and then applied the “ComBat” method in the R package “SVA” for batch correction. Among the datasets related to AD, since the series matrix of GSE33000 was a standardised dataset, the other four datasets were standardised separately. We then combined the 5 standardised datasets and applied the “ComBat” method to perform batch correction.

Differential expression gene analysis of the corrected datasets was performed using the R package “limma”. For the AD datasets, we used a threshold of *p*-value < 0.05, with | log2 (FC) | > 0 for upregulated genes and log2 (FC) < 0 for downregulated genes. For PD datasets we used *p*-value < 0.05, log2 (FC) ≥ 0.5 for upregulated genes and log2 (FC) < =0.5 for downregulated genes.

### Identification of crosstalk genes

The differentially expressed genes of AD and PD were intersected and the shared genes were regarded as potential crosstalk genes. Functional enrichment analysis of the crosstalk genes was performed using “clusterProfiler” (GO Biological processes and KEGG pathways, at a threshold of *p*-value < 0.05.

### Crosstalk genes’ protein-protein interaction (PPI) network analysis

We downloaded protein-protein related gene pairs from MINT (http://mint.bio.uniroma2.it/mint/Welcome.do), HPRD (http://www.hprd.org/index_html), BIOGRID (http://thebiogrid.org/), DIP (http://dip.doe-mbi.ucla.edu/dip/Main.cgi), mentha (http://mentha.uniroma2.it/index.php), PINA (http://cbg.garvan.unsw.edu.au/pina/), InnateDB (http://www.innatedb.com/), and Instruct (http://instruct.yulab.org/index.html) databases. Next, PPI relationship pairs for the crosstalk genes were extracted, and a PPI network was constructed using Cytoscape software, with the plug-in “NetworkAnalyzer” to analyse the network’s topological properties.

### Crosstalk genes’ immune cell enrichment analysis

XCell (https://xcell.ucsf.edu/) was used for cell type enrichment of the crosstalk genes. XCell includes 64 cell types involving multiple adaptive and innate immune cells, hematopoietic progenitors, epithelial cells, and extracellular matrix cells, comprising 48 tumor microenvironment-related cells. We first extracted Case samples from AD and PD datasets and obtained the expression values of crosstalk genes. The gene number limit for the raw enrichment analysis method in the xCell package was reset and the scores for immune-infiltrating cells corresponding to the samples were calculated. Next, “transform scores” and “spill over” were used to obtain the final corrected immune infiltrating cell scores.

### Analysis of immune-related genes and infiltrating immune cells

Immune-related genes were downloaded from an earlier publication (Charoentong et al., 2017) (782 genes, including 431 genes related to 15 adaptive immune cell types and 351 genes related to 13 innate immune cell types) and combined with immune-related genes from Innate DB (https://innatedb.com/annotatedGenes.do?type=innatedb) and ImmPort (https://www.immport.org/home) datasets. The expression values of these immune-related genes in the AD and PD case samples were extracted and the xCell algorithm was used to obtain the expression scores of infiltrating immune cells. The differences between the immune cell fractions in the two diseases was tested using the Wilcoxon’s test (*p* < 0.05).

### Consensus cluster analysis of AD and PD samples based on immune-related genes

We applied consensus clustering to the expression matrix profiles of immune-related genes in AD and PD each, using the “ConsensusClusterPlus” package. Average silhouette width, gap statistic, and the elbow method were used to determine the optimal number of clusters. Next, the samples were clustered using clustering consistency.

### Adaptive and innate immune cell bias analysis

We combined the immune cell fractions and sample clusters of case samples from AD and PD. For each consensus cluster, statistics for cluster distribution of xCell scores for adaptive and innate immune-related genes were computed. The difference between scores of immune cells in different clusters was tested using the Kruskal Wallis test. We also noted the overall scores of immune cells in different clusters to determine immune cells that characterized a cluster.

### Identification of potential biomarkers using LASSO logistic regression

We extracted the expression values of the crosstalk genes for the case and control groups and applied LASSO logistic regression. From the screened crosstalk genes, those common to AD and PD were considered biomarker crosstalk genes. Next, adaptive immune cell-related genes were identified based on the literature, and their expression profiles in AD and PD datasets were screened using LASSO logistic regression. The intersecting genes were recorded as biomarker adaptive immune genes. For Innate immune cell-related genes, we combined those obtained from literature with those obtained from the InnateDB dataset and obtained 1,335 innate immune-cell related genes. LASSO logistic regression was similarly applied, and biomarker innate immune genes were identified. In the next step, the intersections of biomarker crosstalk genes with biomarker adaptive immune cell-related genes and biomarker innate immune cell-related genes were determined. Receiver operating curve (ROC) analysis was performed using these genes’ expression values. Human KEGG pathways and related genes were obtained from the KEGG database (https://www.kegg.jp/) and pathways that correspond to these intersecting genes were identified and all genes in each such pathway were listed. Interactions between the KEGG pathways, biomarker crosstalk genes, biomarker adaptive immune-cell related genes, and biomarker innate immune cell-related genes were identified.

## Results

### Differentially expressed genes

**FIGURE 1:**
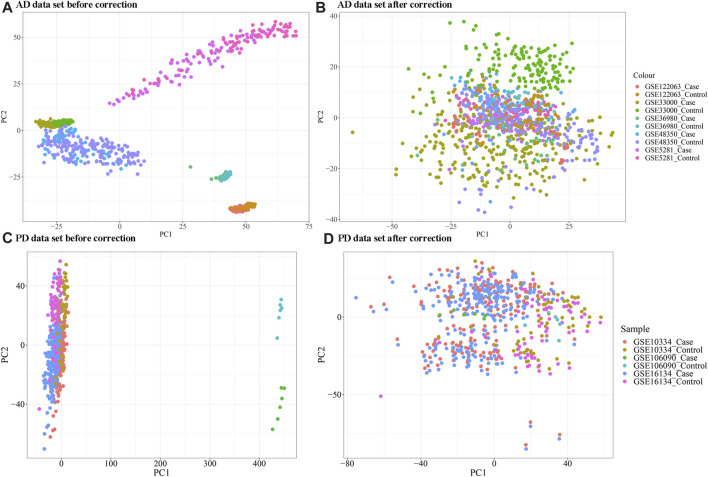
**(A, B)** Principal coordinate analysis (PCA) plot of AD samples clustering before and after batch correction; **(C, D)** PCA analysis plot of PD samples clustering before and after correction. The “ComBat” method in the R package “SVA” was used for batch correction.

**FIGURE 2:**
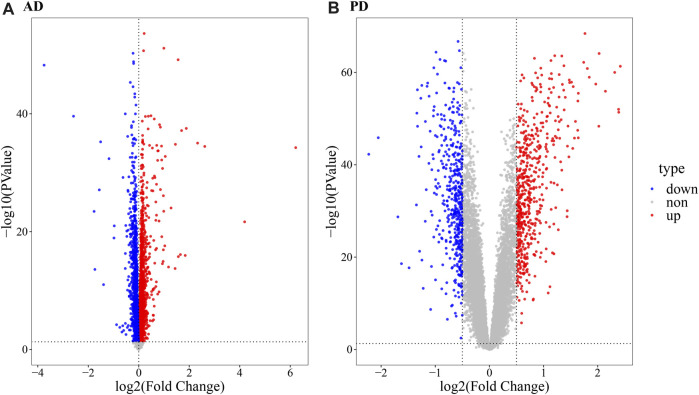
Volcano map depicting differentially expressed genes. **(A)** Volcano map ofdifferentially expressed genes in AD; **(B)** Volcano map of differentially expressed genes in PD. The R package “limma” was used for differential gene expression analysis. For the AD datasets, a threshold of *p*-value < 0.05,| log2 (FC) | > 0 for upregulated genes and log2 (FC) < 0 for downregulated genes was used. For PD datasets, a *p*-value < 0.05, log2 (FC) ≥ 0.5 for upregulated genes and log2 (FC) < = 0.5 for downregulated genes was used.

As evident in Figure 1, significant clustering by batch was noted for both AD and PD gene expression datasets before correction and was reduced post batch correction (Figure 1). Using the batch corrected data, we obtained 4,398 differentially expressed genes in AD and 1,041 differentially expressed genes in PD, respectively. A volcano map was used to display the distribution of the differentially expressed genes (Figure 2).

### Crosstalk genes enrichment in immune related pathways

**FIGURE 3:**
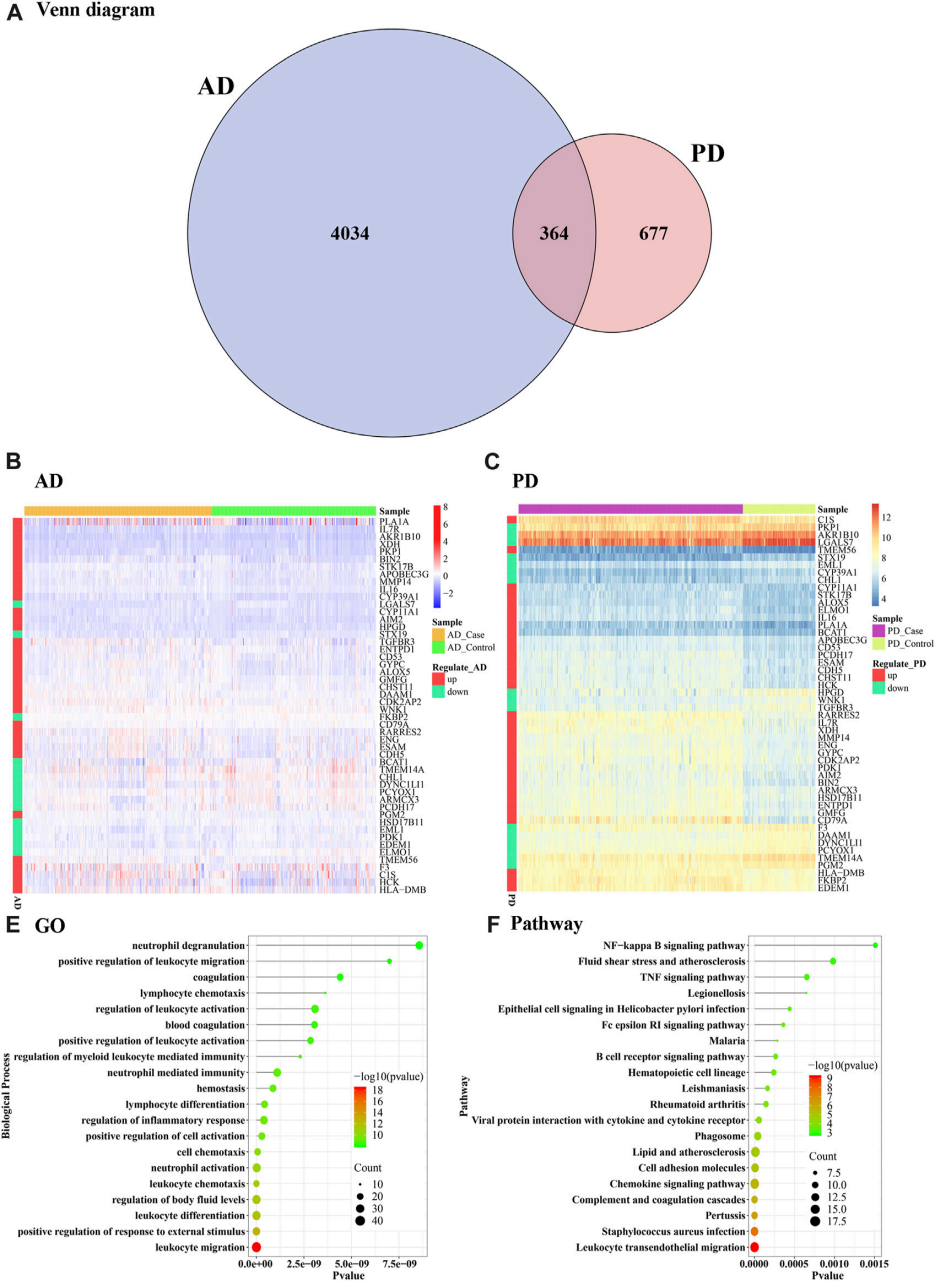
Crosstalk genes and functional enrichment analysis. **(A)** Venn diagram of differentially expressed genes obtained from AD and PD **(B, C)** Heat maps showing the expression levels of the cross talk genes in AD and PD **(D)** Top 20 enriched biological processes in the crosstalk genes; **(E)** KEGG pathways significantly enriched in the crosstalk genes. The top 50 crosstalk genes were used as input for the visualizations.

A total of 364 Crosstalk genes were obtained (Figure 3A) by intersection of the differentially expressed genes of PD and AD. To visualize changes in the expression values of crosstalk genes in different sample types, heat maps were plotted using the “pheatmap” R package, using top 50 Crosstalk genes as the input (Figures 3B, C). To further analyze the functions of the crosstalk genes functional enrichment analysis was performed and significantly enriched GO Biological process and KEGG Pathways were identified and the top 20 were visualized (Figures 3D–E).

Gene ontology analysis showed that the crosstalk genes mainly regulated several leukocyte functions including chemotaxis, migration, differentiation, and myeloid leukocyte related immunity. In particular, neutrophil activation, degranulation, and associated immunity. Blood coagulation, hemostasis and body fluid balance regulation were also enriched among the crosstalk genes (Figure 3D). Among the enriched KEGG pathways, leukocyte transendothelial migration, *S. auerus* infection, and complement and coagulation cascades showed the top-most significance. Innate immune pathways including chemokine signaling, NF kappa beta signaling, and TNF signaling pathways were noted. Lipid and atherosclerosis pathway, epithelial cell signaling and cell-mediated immune pathway B cell receptor signaling were also notably enriched among others including rheumatoid arthritis, Fc epsilon R1 signaling and viral protein interaction with cytokine receptor (Figure 3E).

### Key crosstalk genes identified through PPI network analysis

**FIGURE 4:**
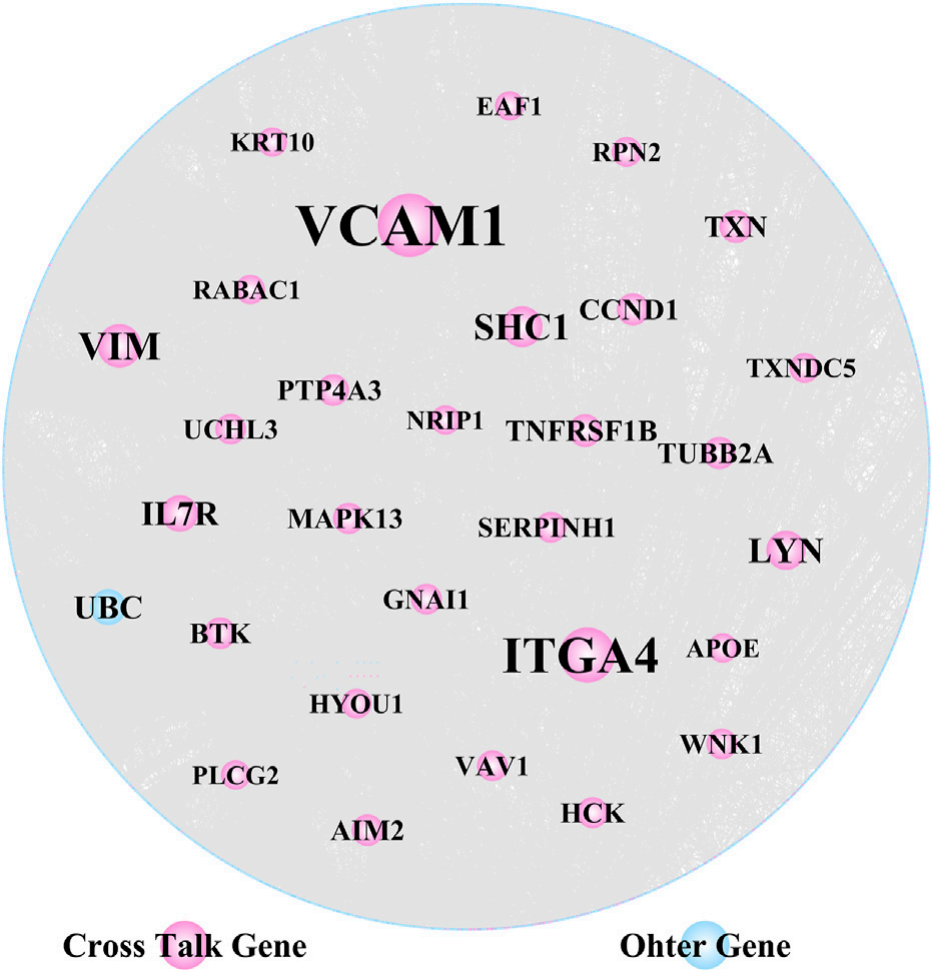
Crosstalk genes’ protein-protein interaction (PPI) network analysis (30 hub nodes obtained from the topological analysis are displayed).

We extracted PPI relationship pairs for the 364 crosstalk genes and constructed a PPI network (Figure 4), which showed 4,870 nodes and 9,657 edges. The topological properties of the network were analyzed, and the top 30 hub node genes (Table 2) were identified based on the degree of gene connectivity and considered as the most important genes nodes in the protein interaction network relationship.

**TABLE 2:** Topological characteristics of the top 30 gene nodes in the crosstalk-gene PPI network.

Name	AD_PD	Degree	Average shortest path length	Betweenness centrality	Closeness centrality	Clustering coefficient	Topological coefficient
VCAM1	up_up	673	2.612737	0.150601	0.38274	0.002503	0.003982
ITGA4	up_up	528	2.611707	0.099121	0.382891	0.003996	0.004326
VIM	up_up	320	2.645095	0.105724	0.378058	0.003174	0.006497
SHC1	up_up	273	2.812861	0.065675	0.35551	0.005602	0.009555
LYN	up_up	243	2.731451	0.072094	0.366106	0.006462	0.007585
IL7R	up_up	199	2.86892	0.035555	0.348563	0.002335	0.010105
UBC		184	2.253504	0.340648	0.443753	0.005702	0.010612
TXN	down_down	132	2.796785	0.027568	0.357553	0.0096	0.011983
TNFRSF1B	up_up	131	2.958368	0.032291	0.338024	0.001174	0.011297
TUBB2A	up_down	124	2.865829	0.030234	0.348939	0.003278	0.012209
CCND1	up_down	121	3.020816	0.02919	0.331036	0.00124	0.017982
BTK	up_up	112	2.934048	0.022542	0.340826	0.015766	0.017501
AIM2	up_up	109	3.370363	0.02737	0.296704	0	0.016514
PTP4A3	up_up	109	3.336974	0.024112	0.299673	1.70E-04	0.017308
MAPK13	up_down	103	3.092745	0.013791	0.323337	0	0.024619
HCK	up_up	100	2.968879	0.018268	0.336827	0.012525	0.020322
SERPINH1	up_up	96	2.955894	0.020851	0.338307	0.003728	0.017841
VAV1	up_up	95	2.930338	0.014357	0.341258	0.028219	0.019849
GNAI1	down_down	93	3.09357	0.029624	0.323251	0.001636	0.015197
WNK1	up_down	92	3.109439	0.022769	0.321601	4.78E-04	0.022952
UCHL3	down_down	90	3.072135	0.018117	0.325507	0.002747	0.021144
HYOU1	down_up	80	3.079555	0.015011	0.324722	0.001266	0.02724
RPN2	up_up	79	3.097486	0.01577	0.322843	0	0.027241
TXNDC5	up_up	78	3.068219	0.019356	0.325922	0	0.025968
RABAC1	down_up	71	3.022465	0.01991	0.330856	0.002414	0.016747
APOE	up_up	70	2.906018	0.020983	0.344113	0.004555	0.016556
PLCG2	up_up	69	2.955482	0.010744	0.338354	0.047315	0.024349
KRT10	up_down	68	2.854699	0.00891	0.3503	0.04302	0.027171
NRIP1	down_down	68	3.084295	0.015047	0.324223	0	0.027749
EAF1	down_down	67	3.571517	0.019232	0.279993	0.003618	0.02098

VCAM1, ITGA4 and VIM, were noted as the top genes playing an important role in the network, and were upregulated in both AD and PD. Several of the gene nodes showed opposing patterns of regulation in the two diseases. KRT10, WNK1, MAPK13, TUBB2A and CCND1 were upregulated in AD but downregulated in PD. Conversely, RABAC1 and HYOU1 were downregulated in AD but upregulated in PD.

### Immune cell fractions enriched by the crosstalk genes show comparative differences between AD and PD

**FIGURE 5:**
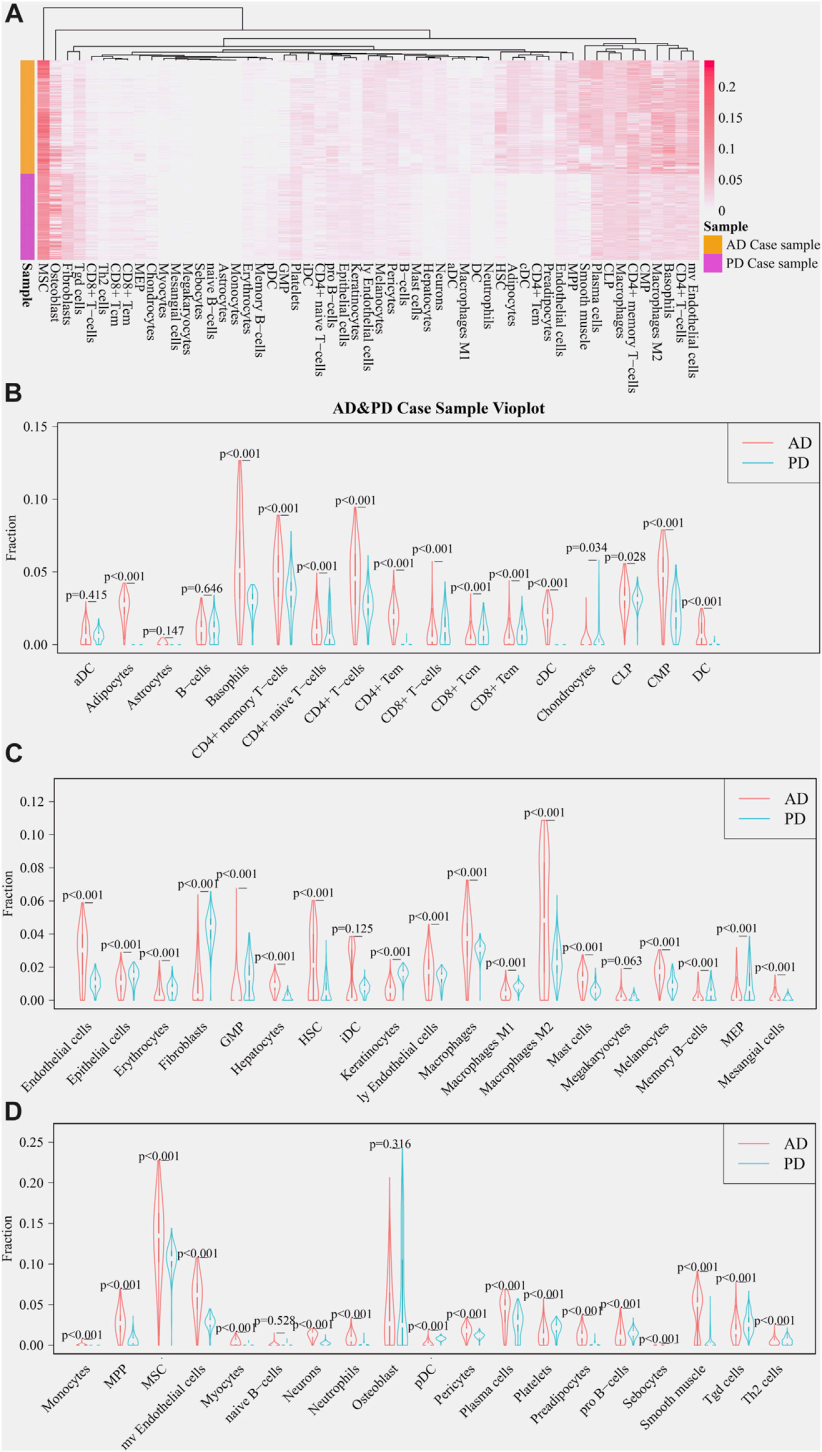
Expression of immune cells in AD and PD. **(A)** Thermographic representation of immune cell expression scores in AD and PD. **(B–D)** Violin diagram of immune cells expression scores in AD and PD. The “xCell” package was used to obtain scores of 55 immune infiltrating cells in both disease sample datasets and differences were tested using Wilcoxon’s analysis.

Using the xCell package, scores of immune-infiltrating cells corresponding to the 364 Crosstalk genes were calculated and “transform scores” and “spillOver” were applied to obtain the final corrected immune cell scores. Scores of 55 immune cell types in the AD and PD datasets were noted and a heatmap was used to display the scores of immune infiltrating cells in AD and PD datasets (Figure 5A).

A violin diagram drawn using “vioplot” was used to depict the scores of each immune infiltrating cell in both diseases (Figures 5B–D). The difference in scores of immune infiltrating cells for the Case samples of AD and PD datasets was tested using Wilcoxson’s test. The cells were grouped in three categories and displayed (Figures 5B–D). We can see that several immune cell types are closely related in both diseases. AD samples showed highly significantly higher scores for adipocytes, CD4^+^ and CD8^+^ T-cell subsets, cDC and DC cell types. Endothelial cells, mast cells and macrophage cells, along with neutrophils, MSC, Th2, HSC, iDC, plasma cells and pro-B cells were comparatively overexpressed in AD, in particular, M2 macrophage cells. Chondrocytes, osteoblasts and fibroblast cells showed greater overexpression in PD.

**FIGURE 6:**
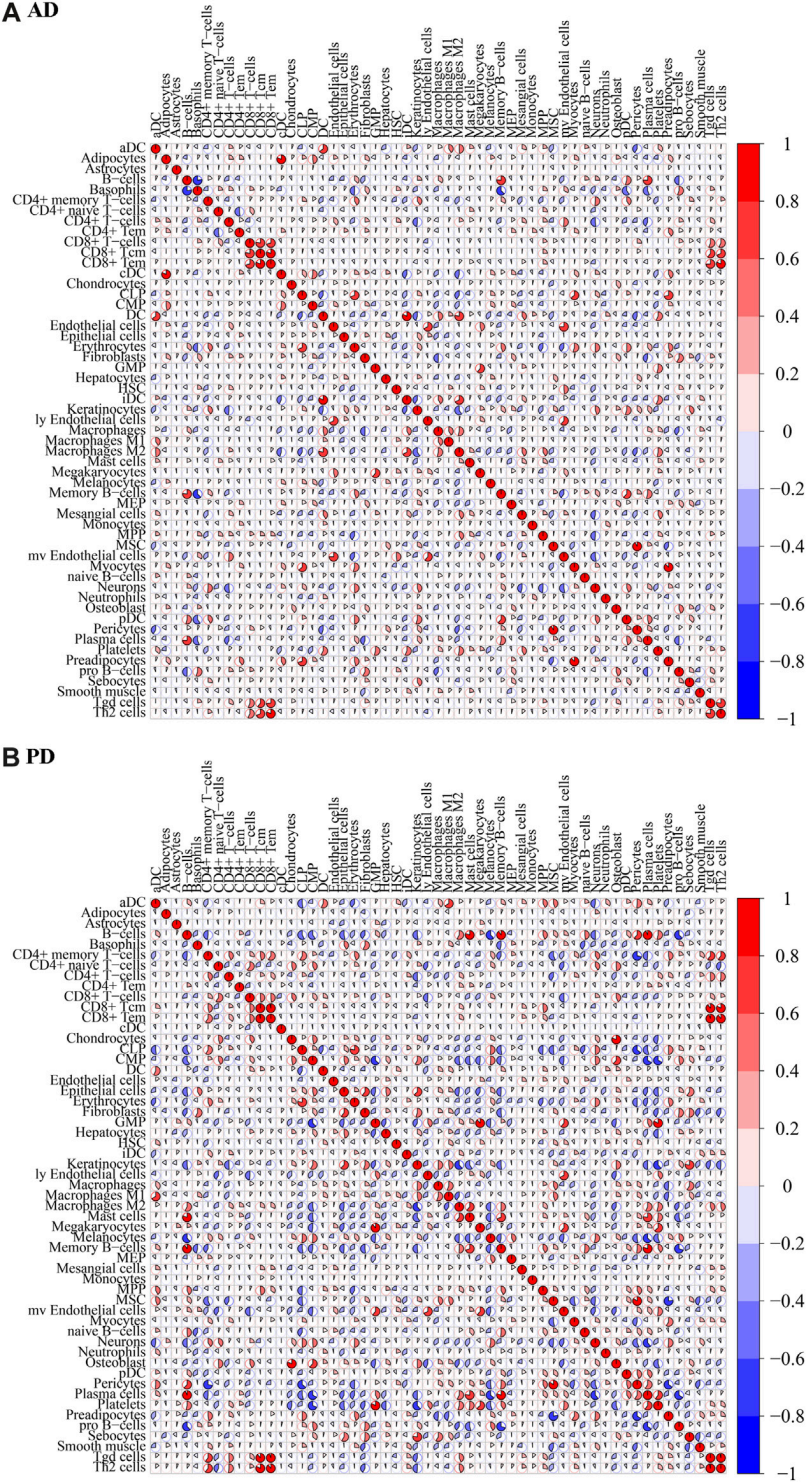
Correlation of immune cell scores in Case groups of **(A)** AD and **(B)** PD. xCell scores for each immune cell type in the case samples were subjected to Spearman’s correlation analysis.

To test the correlation between immune cells, a correlation analysis of xCell scores for Case samples for each immune cell type in the two data sets was applied (Figures 6A, B).

For AD, immune cell CD8^+^ Tem and Th2 cells were highly positively correlated (COR = 0.8196), CD8^+^ Tcm and CD8^+^ Tem were highly positively correlated (COR = 0.8127), CD8^+^ T-cells and CD8^+^ Tcm were highly positively correlated (COR = 0.7822). B-cells were highly negatively correlated with Basophils (COR = −0.7763). For PD, CD8^+^ Tcm and CD8^+^ Tem were highly positively correlated (cor = 0.9695), Tgd cells and Th2 cells were highly positively correlated (cor = 0.9657), CD8^+^ Tem and Tgd cells were highly positively correlated (0.9500), CD8^+^ Tem and Th2 cells were highly positively correlated (cor = 0.8954). MSC and Preadipocytes were highly negatively correlated (cor = −0.7754), CD4^+^ memory T-cells and Pericytes were highly negatively correlated (cor = −0.7173), Memory B-cells and pro B-cells were highly negatively correlated (cor = −0.6979).

### Immune-related genes and immune cells enriched in AD and PD

**FIGURE 7:**
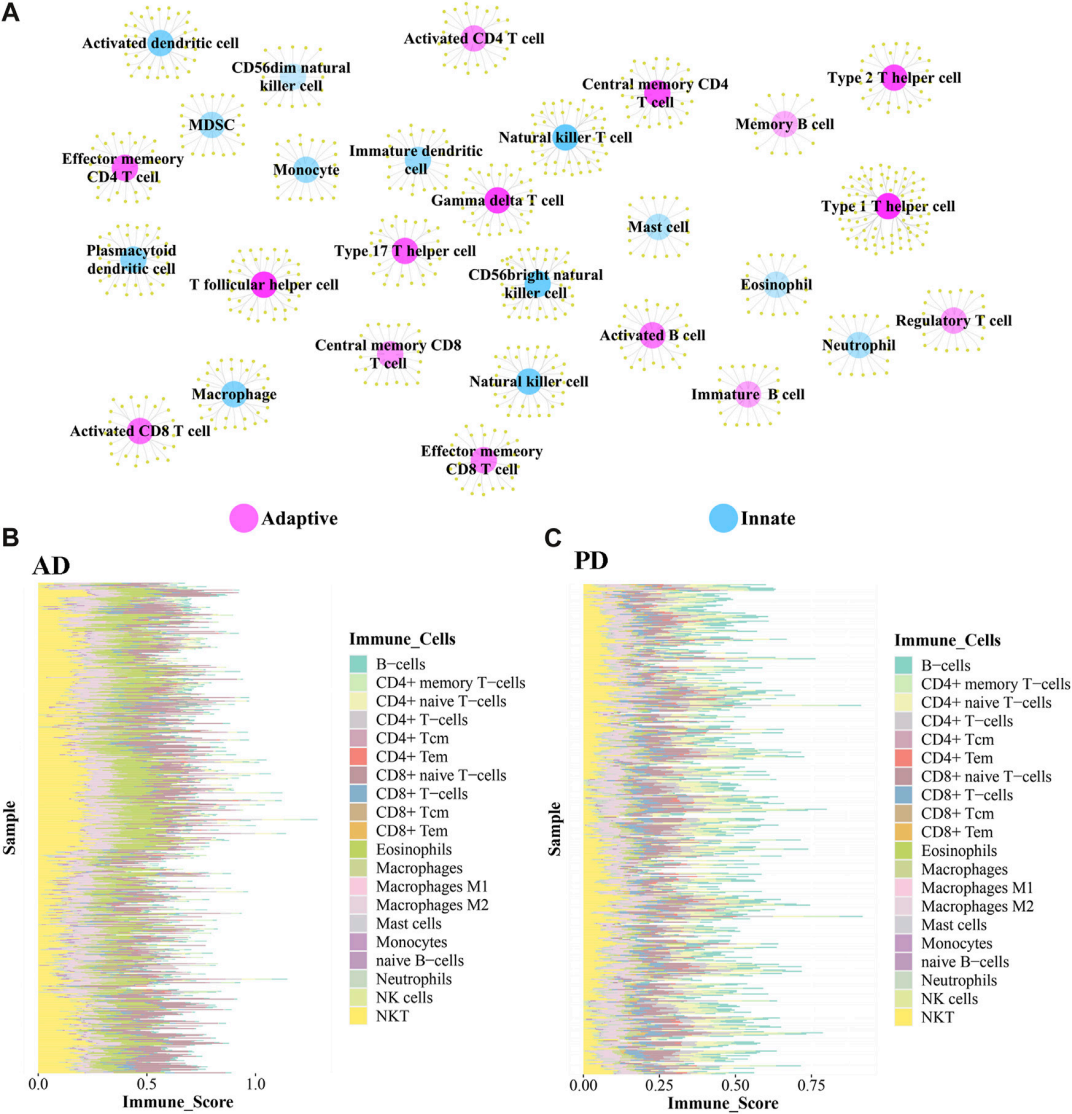
**(A)** Adaptive immune gene-associated immune cells and Innate gene-associated immune cells. **(B, C)** Expression levels of 20 Adaptive and Innate immune cells in AD and PD.

The immune-related genes downloaded from the literature included genes related to 15 Adaptive immune cell and 13 Innate immune cell types (Figure 7A). Further immune-related genes were obtained from InnateDB and ImmPort databases. We merged the immune-related genes acquired from literature, InnateDB, and ImmPort databases to obtain 3,046 immune genes as the final immune-related gene dataset. We extracted the expression values of these 3,046 immune genes in the Case samples of AD and PD and found that 1,142 immune genes were expressed in AD whereas 2,396 immune genes were expressed in PD. Using the xCell algorithm, we obtained the expression scores of immune cells in the Case samples of AD and PD. Since the names of 64 cell types included in xCell were different from the names of 28 cell types listed in literature, we identified and listed the cell types (Table 3).

**TABLE 3:** Common immune cells xCell and literature (Charoentong et al., 2017).

[xCell] cells 1	[xCell] cells 2	[Charoentong et al., 2017]Cells	[Charoentong et al., 2017] cells immunity
aDC	iDC	Activated B cell	Adaptive
Adipocytes	Keratinocytes	Activated CD4 T cell	Adaptive
Astrocytes	ly Endothelial cells	Activated CD8 T cell	Adaptive
B-cells	Macrophages	Central memory CD4 T cell	Adaptive
Basophils	Macrophages M1	Central memory CD8 T cell	Adaptive
CD4^+^ memory T-cells	Macrophages M2	Effector memeory CD4 T cell	Adaptive
CD4^+^ naive T-cells	Mast cells	Effector memeory CD8 T cell	Adaptive
CD4^+^ T-cells	Megakaryocytes	Gamma delta T cell	Adaptive
CD4^+^ Tcm	Melanocytes	Immature B cell	Adaptive
CD4^+^ Tem	Memory B-cells	Memory B cell	Adaptive
CD8^+^ naive T-cells	MEP	Regulatory T cell	Adaptive
CD8^+^ T-cells	Mesangial cells	T follicular helper cell	Adaptive
CD8^+^ Tcm	Monocytes	Type 1 T helper cell	Adaptive
CD8^+^ Tem	MPP	Type 17 T helper cell	Adaptive
cDC	MSC	Type 2 T helper cell	Adaptive
Chondrocytes	mv Endothelial cells	Activated dendritic cell	Innate
Class-switched memory B-cells	Myocytes	CD56bright natural killer cell	Innate
CLP	naive B-cells	CD56dim natural killer cell	Innate
CMP	Neurons	Eosinophil	Innate
DC	Neutrophils	Immature dendritic cell	Innate
Endothelial cells	NK cells	Macrophage	Innate
Eosinophils	NKT	Mast cell	Innate
Epithelial cells	Osteoblast	MDSC	Innate
Erythrocytes	pDC	Monocyte	Innate
Fibroblasts	Pericytes	Natural killer cell	Innate
GMP	Plasma cells	Natural killer T cell	Innate
Hepatocytes	Platelets	Neutrophil	Innate
HSC	Preadipocytes	Plasmacytoid dendritic cell	Innate
Smooth muscle	pro B-cells		
Tgd cells	Sebocytes		
Th1 cells	Skeletal muscle		
Th2 cells	Tregs		

10 adaptive and 10 innate immune cell types were noted in xCell and are marked with different colors. These 20 cell types were extracted for subsequent analysis and their scores were analysed. The fraction of these immune cells is depicted in Figure 7B.

Among the adaptive immune cells, most cells showed higher expression in AD, while CD4^+^ naive T−cells and B−cells were highly expressed in PD samples as compared with AD samples. The expression of Eosinophils was higher in AD disease samples than in PD disease samples. Macrophages M2, Natural killer T cell (NKT) and CD8^+^ naive T-cells were highly expressed in AD and PD samples. Macrophages M2 and Natural killer T cell (NKT) are innate immune cells, while CD8^+^ naive T-cells are adaptive.

**FIGURE 8:**
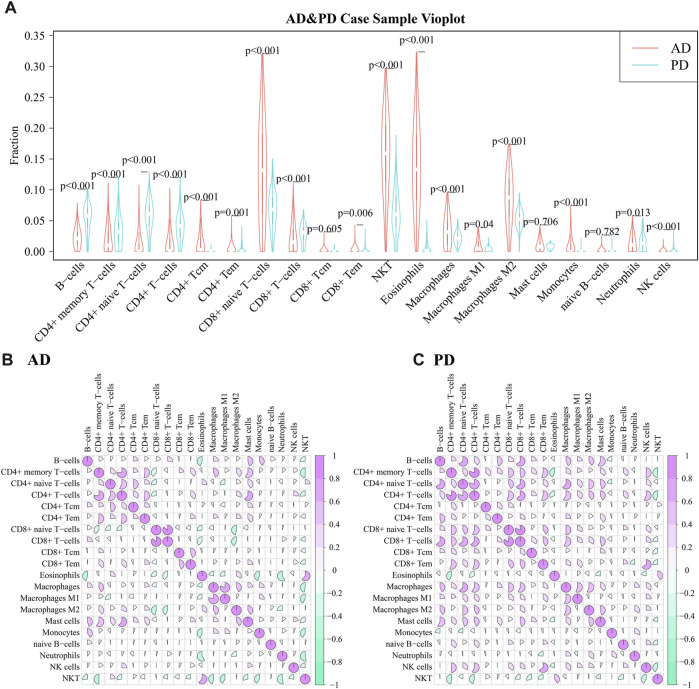
**(A)** Comparison of scores of 20 immune infiltrating cell scores in AD and PD; **(B)** Correlation between infiltrating immune cell scores in AD; Differences were tested using Wilcoxon’s analysis. **(C)** Correlation between infiltrating immune cell scores in PD. Spearman’s correlation analysis was performed.

A violin diagram was drawn to depict the scores of each immune infiltrating cell in both diseases (Figure 8A) and differences between AD and PD datasets were tested using Wilcoxon’s test (*p* < 0.05). Macrophages M2, Natural killer T cell (NKT) and CD8^+^ naive T-cells were found to be significantly different in AD and PD. Then, we examined the correlation between the 20 immune cells in AD and PD (Figures 8B, C). CD8^+^ T-cells and CD8^+^ naive T-cells, CD4^+^ memory T-cells and CD4^+^ T-cells were highly positively correlated in AD and PD (Table 4).

**TABLE 4:** 20 types of immune cells highly positively correlated (COR ≥0.6) in AD and PD.

Cell1	Cell2	PD_cor	PD_*p*-value
CD8^+^ naive T-cells	CD8^+^ T-cells	0.81793	8.186E-105
CD4^+^ memory T-cells	CD4^+^ T-cells	0.801068	1.9407E-97
CD4^+^ naive T-cells	CD4^+^ T-cells	0.799333	1.0145E-96
B-cells	CD4^+^ naive T-cells	0.660739	2.7763E-55
CD8^+^ Tem	NK cells	0.654085	7.5844E-54
CD4^+^ naive T-cells	CD8^+^ T-cells	0.652631	1.5451E-53
B-cells	CD8^+^ T-cells	0.643272	1.3744E-51
CD4^+^ T-cells	CD8^+^ T-cells	0.633225	1.433E-49
CD4^+^ naive T-cells	Macrophages	0.628991	9.6533E-49
Cell1	Cell2	AD_cor	AD_*p*-value
CD8^+^ naive T-cells	CD8^+^ T-cells	0.854951	6.467E-163
Macrophages	Macrophages M1	0.796427	2.673E-125
CD4^+^ memory T-cells	CD4^+^ T-cells	0.767739	4.144E-111
CD4^+^ T-cells	Mast cells	0.62343	2.8898E-62
Eosinophils	NKT	0.620524	1.5309E-61

### Consensus cluster analysis of immune cells based on immune genes

**FIGURE 9:**
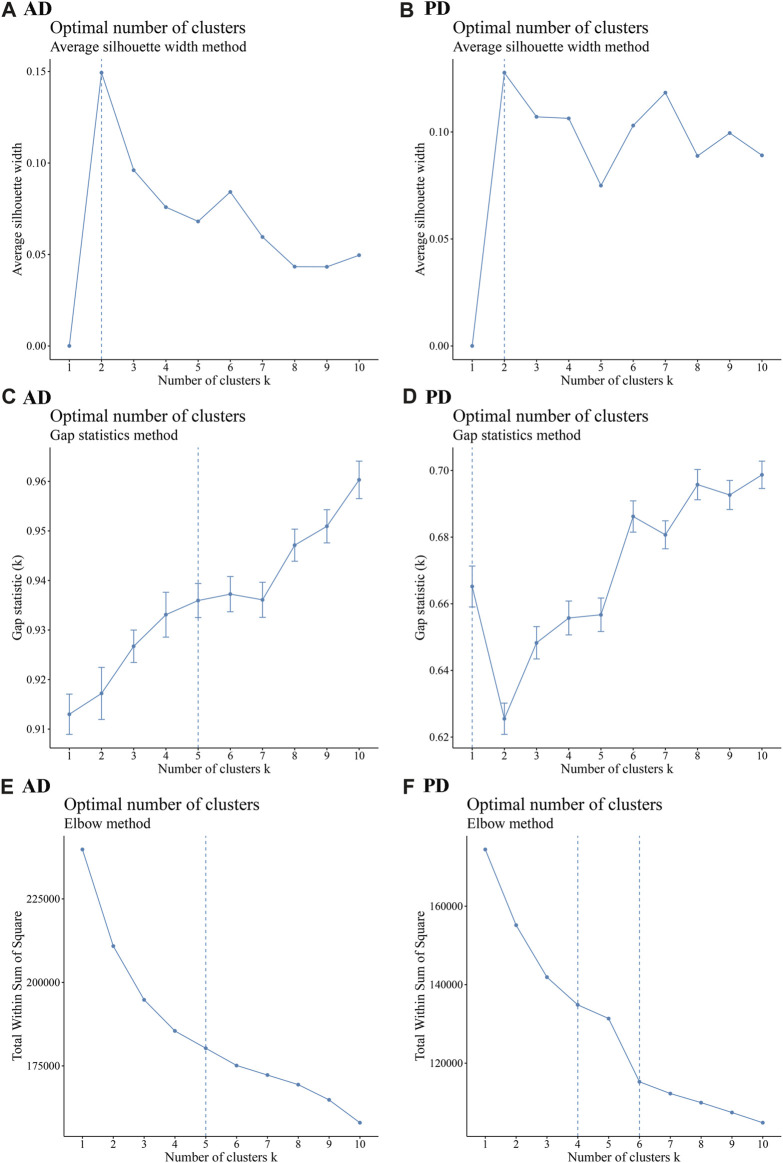
Cluster number analysis using different methods **(A–C)** Average silhouette width, Gap statistic, and Elbow method to analyse the number of AD clusters; **(D–F)** Average silhouette width, Gap statistic, and Elbow method to analyse the number of PD clusters. The “ConsensusClusterPlus” R package was applied for cluster analysis.

1,142 immune genes found expressed in AD and 2,396 immune genes found expressed in PD were subjected to Consensus Clustering. The maxK values were determined using average silhouette width, gap statistic, and the elbow method to find the optimal number of clusters for the AD and PD expression matrices (Figures 9A–F).

**FIGURE 10:**
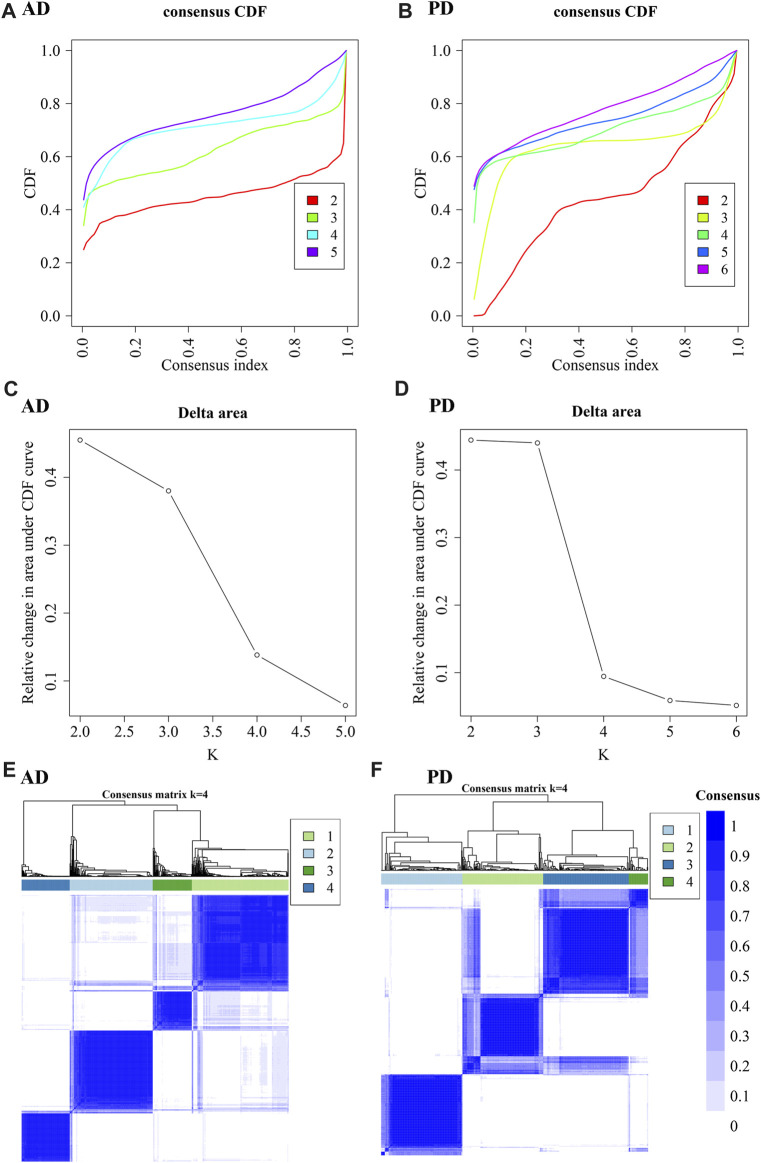
Cluster consistency analysis. **(A, B)** Consistent cumulative distribution function (CDF) plots for AD and PD. This figure shows the cumulative distribution function of scores with different values of K, which is used to determine the approximate maximum value of CDF for a selected k value, and the cluster analysis result that is the most reliable. That is, the k value with a small descending slope of CDF is considered. **(C, D)** Delta Area Plot of AD and PD: This figure shows the relative changes of areas under the CDF curve compared to k and k-1. When k = 2, since there is no k = 1, the first point represents the total area under the CDF curve at k = 2 (that is, the area of the center line in Figure AB), rather than the relative change in area. **(E, F)** Consistent clustering diagram of AD and PD.

As seen in Figure 9, the number of optimal clusters denoted by the three methods were different, which may be related to the large number of gene features and the differences in the algorithms. In AD, the maximum number of clusters was 5 and the minimum number was 2. In PD, the maximum number was 6 and the minimum number was 1. Clustering consistency results for 2-5 clusters in AD, and 2-6 clusters in PD were analysed. Key clustering consistency results for AD and PD are depicted in Figures 10A–F.

**FIGURE 11:**
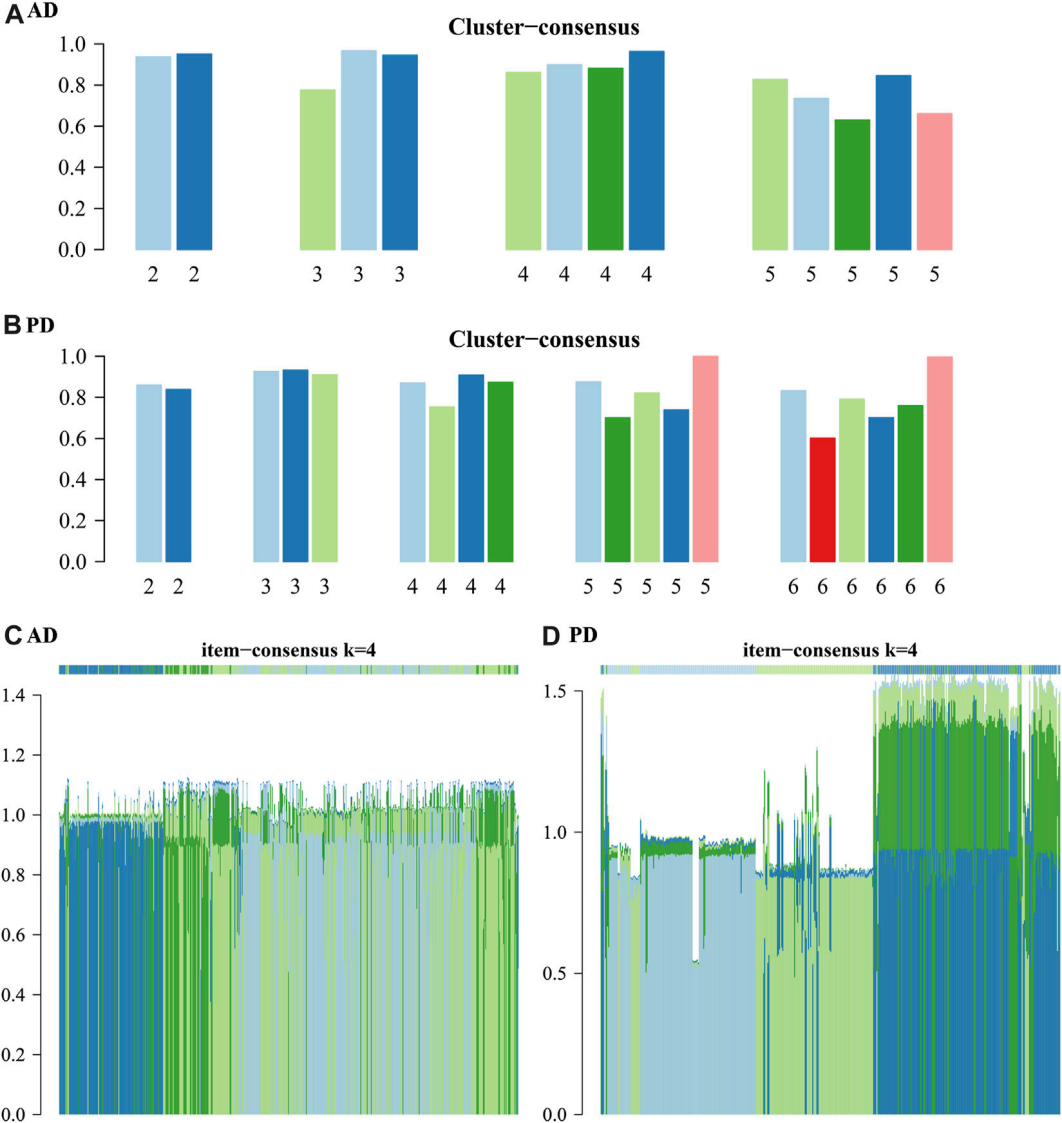
Cluster-consensus and item-consensus for AD and PD. **(A–B)** Cluster-Consensus Plot for AD and PD. These figures show the cluster-consensus value of each cluster under AD and PD (The mean value of pairwise consensus values of members in the cluster). The higher the value, the higher the stability. It can be used to assess the cluster-consensus values under the same and between different k values. We can see in **(A)**, for AD, when k = 4, the mean values are high. In **(B)**, for PD, when k = 4, the mean values are also very high. **(C, D)** Item-consensus Plot for AD and PD: This figure shows the score of each sample for AD and PD when k = 4.

The results show that to get the final k value, the descending slope of the Central Line and the relative change of the area under the CDF curve between K and K-1 should be as small as possible. We finally choose K = 4 for AD and PD both. Figure EF shows the correlation between AD and PD samples at the selected k values. The rows and columns of the matrix represent the samples. Consistency matrix values are shown in white to dark blue on a scale from 0 (impossible to cluster together) to 1 (always cluster together). The consistency matrix is arranged according to the consistency categories (tree at the top of the heatmap). The bar between the tree and the heat map is the category. The more scattered the dark blue squares, the weaker the clustering results. The cluster-consensus and item-consensus for AD and PD was analysed using the calcICL method in the ConsensusClusterPlus package (Figures 11A–D).

From Figure 11 we can see whether the classification of each sample has sufficient fidelity, to help determine the k value. As shown in Figures 10, 11 we clustered the AD and PD disease samples in 4 clusters each. The sample clustering results and the 20 Adaptive and Innate immune cell scores for AD and PD across all samples are shown (Figure 7B). Next, we combined the immune cell fractions and sample cluster results for the Case samples from AD and PD for subsequent analysis.

### Adaptive and innate immune cell bias analysis

**FIGURE 12:**
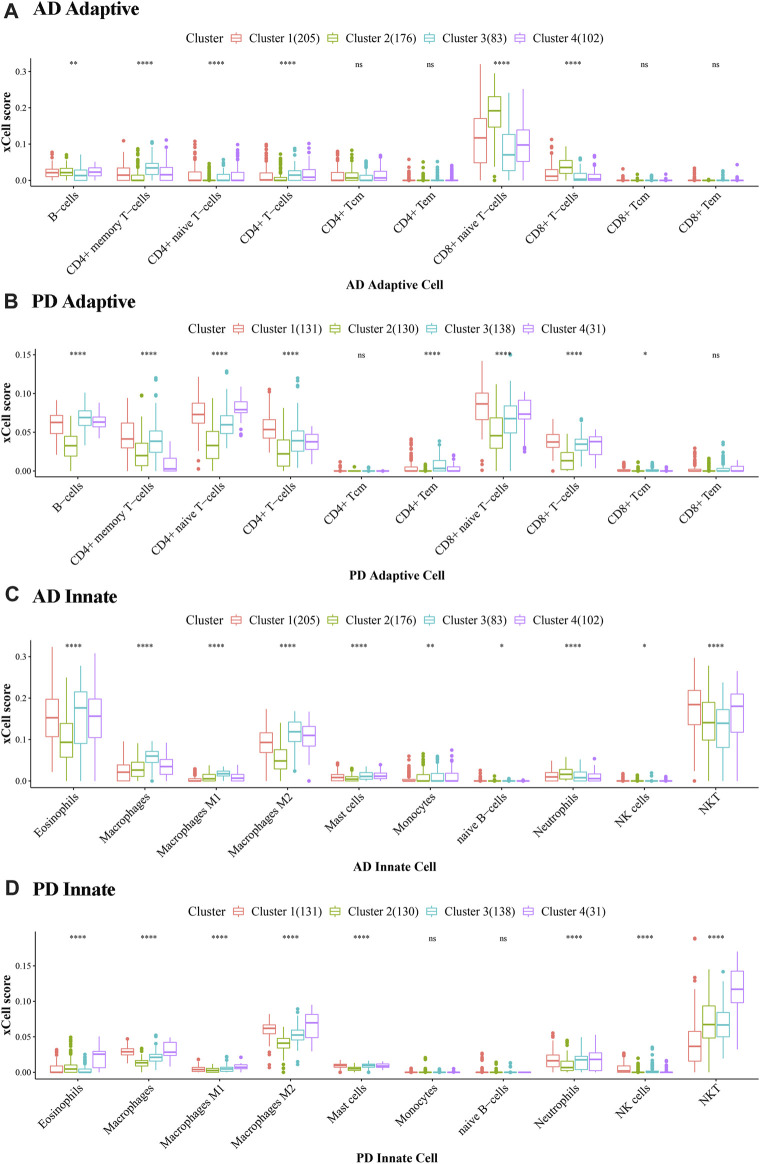
Cluster level expression of Adaptive and Innate immune genes in AD and PD. **(A, B)** Adaptive immune gene expression in AD and PD. **(C, D)** Innate immune gene expression in AD and PD. ns: *p* > 0.05; *: *p* ≤ 0.05; **: *p* ≤ 0.01; ***: *p* ≤ 0.001; ****: *p* ≤ 0.0001. The xCell scores of Adaptive and Innate immune genes in AD and PD were compared using Kruskal. Wallis tests to test differences in scores of immune cells in different clusters.

For each consensus cluster, we calculated the cluster distribution of xCell scores of Adaptive and Innate immune genes in AD and PD and presented these in a box plot (Figures 12A–D). The Kruskal. Wallis test was performed to test differences in scores of immune cells in different clusters.

In Figure 12 we can see that there were significant differences between immune cells in different clusters, and the greater this difference, the more marked the difference between the clusters. We can also see the overall scores of immune cells in different clusters. The top 3 immune cells from the significant clusters in AD and PD were considered high expression immune cells that play an important role in disease pathology (Table 5).

**TABLE 5:** Significant immune cell populations in AD and PD.

		Adaptive immune cells top 3			Innate immune cells top 3		
AD	cluster1	B-cells	CD4^+^ memory T-cells	CD8^+^ naive T-cells	Eosinophils	Macrophages M2	NKT
	cluster2	B-cells	CD8^+^ T-cells	CD8^+^ naive T-cells	Eosinophils	Macrophages M2	NKT
	cluster3	B-cells	CD4^+^ memory T-cells	CD8^+^ naive T-cells	Eosinophils	Macrophages M2	NKT
	cluster4	B-cells	CD4^+^ memory T-cells	CD8^+^ naive T-cells	Eosinophils	Macrophages M2	NKT
PD	cluster1	B-cells	CD4^+^ memory T-cells	CD8^+^ naive T-cells	Macrophage	Macrophages M2	NKT
	cluster2	B-cells	CD4^+^ memory T-cells	CD8^+^ naive T-cells	Macrophage	Macrophages M2	NKT
	cluster3	B-cells	CD4^+^ memory T-cells	CD8^+^ naive T-cells	Macrophage	Macrophages M2	NKT
	cluster4	B-cells	CD4^+^ memory T-cells	CD8^+^ naive T-cells	Macrophage	Macrophages M2	NKT

The results showed that B-cells, CD4^+^ memory T-cells and CD8^+^ naive T-cells were adaptive immune cells that were highly expressed in all 4 clusters of both diseases, and innate immune cells Macrophages M2 and NKT, were similarly highly expressed in all clusters. Adaptive immune cells CD8^+^ naive T− Cells were significantly different between cluster 2 and cluster 3 in AD, and in PD, both.

**FIGURE 13:**
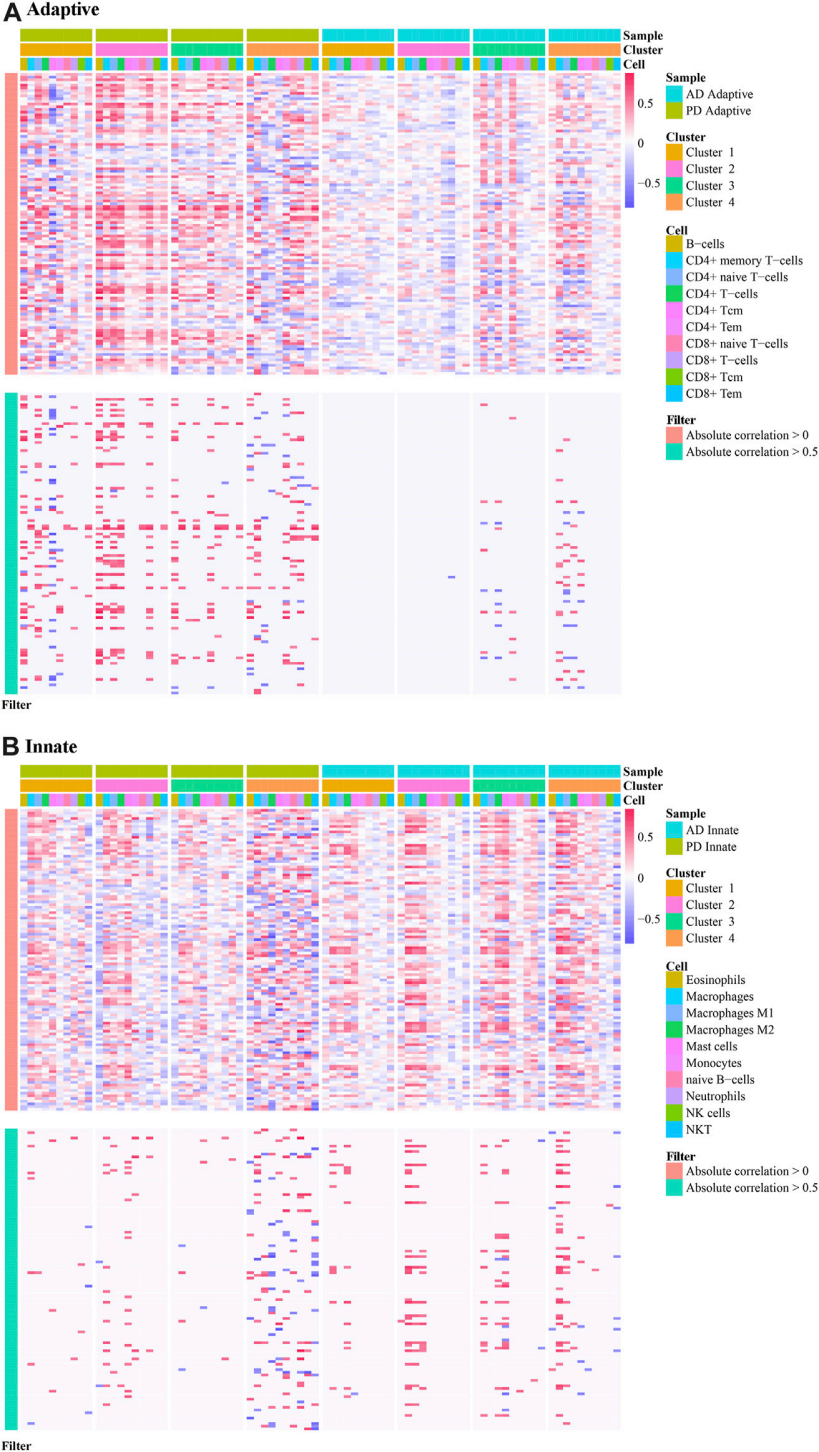
Correlation analysis applied to immune cell scores in different clusters. **(A)** The correlation of adaptive immune-related genes in different clusters in AD and PD; **(B)** The correlation of innate immune-related genes in different clusters in AD and PD. Spearman’s correlation analysis was performed.

We extracted the immune-related genes from the Crosstalk gene dataset and obtained 112 genes in total. Then we extracted the expression values of these 112 genes in the Case samples of AD and PD. Correlation analysis was conducted by combining these values with the xCell scores. These two datasets pertaining to each cluster were subjected to correlation analysis. Correlation results were obtained for each of the 8 clusters and are depicted (Figures 13A, B) allowing an estimation of immune cells bias in the different clusters, and also to allow for selection of specific cluster of subjects for longitudinal study.

The results show immune cell biases in different clusters. For adaptive immune-related genes, cluster1 and cluster4 in PD were highly correlated with a variety of immune cells. In cluster2 and cluster3, immune genes were positively correlated with immune cells. Adaptive immune related genes were highly correlated with a variety of immune cells in cluster 4 of AD, and participated in a variety of immune patterns (Figure 13A). For innate immune related genes, multiple immune cells in cluster4 of PD and cluster4 of AD were highly correlated, suggesting that innate genes were more active in cluster4 samples of PD and AD (Figure 13B).

### Candidate biomarkers identified using LASSO logistic regression

**FIGURE 14:**
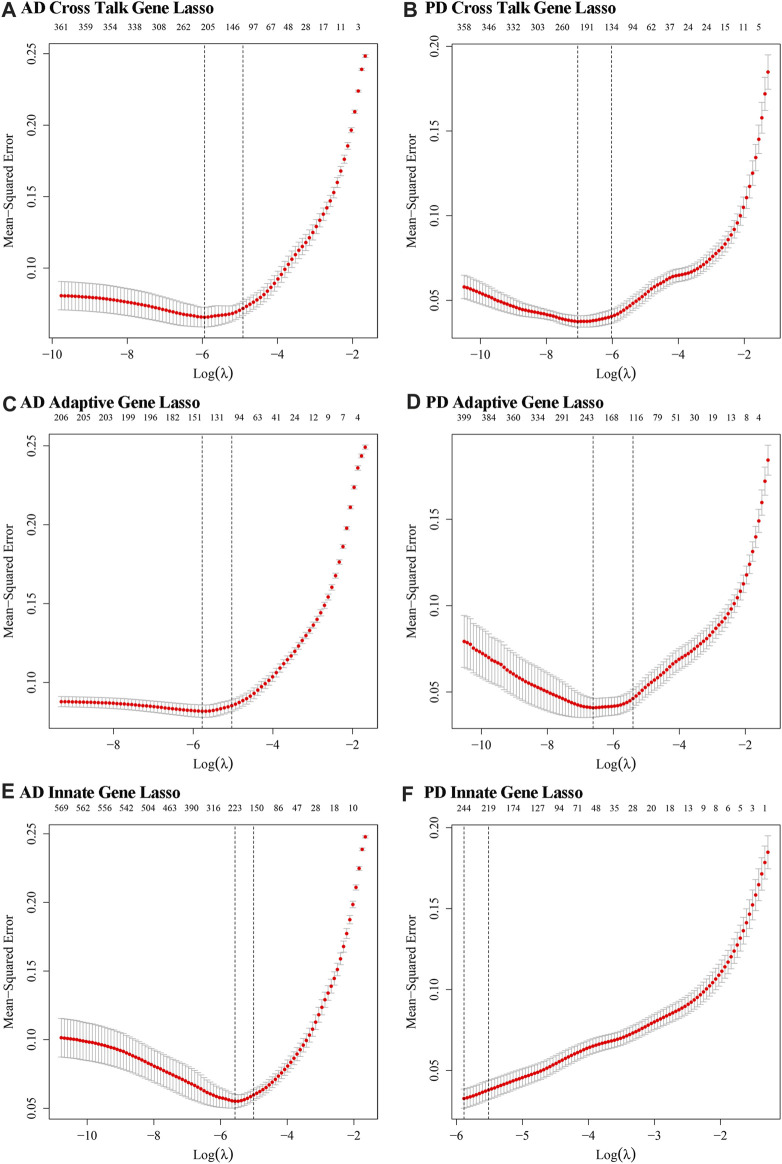
The optimal lambda values obtained from LASSO regression modelling of innate immune genes and adaptive immune genes. **(A, B)** Crosstalk genes: relationship between lambda value and mean square error in AD and PD Lasso regression analysis. The abscissa is log (lambda) and the ordinate is mean square error. There are two dashed lines in the figure, one is the value of λ with the minimum mean square error and the other is the value of λ with the standard error from the minimum mean square error. **(C, D)** Adaptive immune genes: lambda value and mean square error in AD and PD Lasso regression analysis. **(E, F)** Innate immune genes: lambda value and mean square error in AD and PD Lasso regression analysis.

Datasets of expression values for Case and Control samples of AD and PD were obtained. The expression values of the crosstalk genes were subjected to LASSO Logistic Regression to screen the crosstalk genes (Figures 14A, B). The intersecting genes were selected and 127 genes were recorded as the candidate biomarker crosstalk genes.

For Adaptive immune-related genes, we extracted 431 genes’ expression profiles, which included 210 genes found in AD and 408 genes found in PD datasets and applied LASSO Logistic Regression (Figures 14C, D). The intersecting genes among AD and PD were selected and a total of 78 genes were recorded as the biomarker adaptive immune genes.

For Innate immune genes, we combined the innate immune genes obtained from literature with those obtained from InnateDB data to obtain 1,335 Innate immune genes. 571 such genes were found in AD and 1,183 in PD. LASSO Logistic Regression was applied (Figures 14E, F) and a total of 32 intersecting genes were recorded as biomarker Innate immune gene. A diagram displayed the variation of the remaining variables’ gene coefficients with different lambda values from the LASSO regression analysis.

**FIGURE 15:**
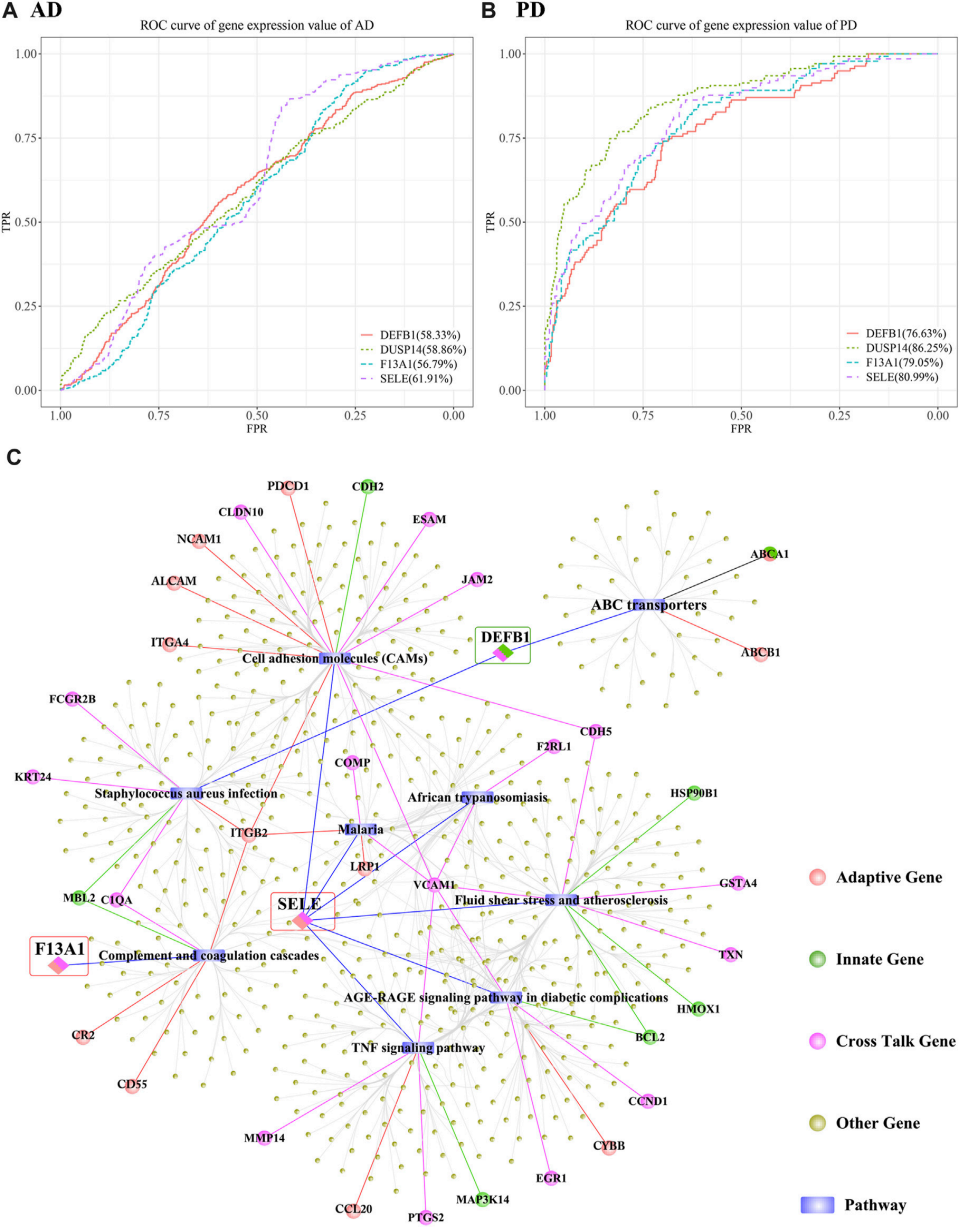
Predictive efficacy of DUSP14, F13A1, SELE and DEFB1 in AD and PD. **(A, B)** ROC results of DUSP14, F13A1, SELE and DEFB1 in AD and PD; **(C)** Functional correlation analysis results of DUSP14, F13A1, SELE and DEFB1.

We obtained 3 genes from the intersection of the biomarker crosstalk genes and the biomarker adaptive immune genes, and 1 gene (DEFB1) from the intersection of the biomarker crosstalk genes with the biomarker innate-immune genes. ROC analysis using the expression values of these 4 genes yielded 3 genes (DUSP14, F13A1, SELE) (Figures 15A, B).

AUC (AUC>70%) values obtained for DUSP14, F13A1, SELE and DEFB1 in discriminating PD were higher than those for AD prediction. SELE performed better than the other 3 genes in discriminating both AD and PD. To further analyse the functions of these genes, we obtained datasets of human KEGG pathways and related genes and mined the corresponding pathways, and then isolated all the genes in each pathway. We examined whether there is interaction between each pathway and the biomarker crosstalk genes, biomarker adaptive immune genes, and biomarker innate immune genes (Figure 15C). The results showed that SELE, an adaptive immune gene, mainly regulates the TNF signalling pathway, cell adhesion molecules (CAMs) and fluid shear stress and atherosclerosis. Within the TNF signalling pathway, VCAM1 represents a specific type of Cell adhesion Molecule (CAM). Within Cell adhesion Molecules (CAMs), the adaptive immune gene ITGB2 regulates both *Staphylococcus aureus* infection and complement and coagulation cascades. From Figure 15C, we can see that F13A1 is mainly involved in the regulation of complement and coagulation cascades pathway. DEFB1, an innate immune-related gene, is mainly involved in the regulation of *S. aureus* infection and ABC transporters. Within *S. aureus* infection, other crosstalk genes such as KRT24 and FCGR2B also participate in the regulation. In addition, ITGB2 and other genes are associated with other pathways to regulate the immune function in both AD and PD. It can be inferred from the above that immune-related crosstalk genes interact with other genes and jointly influence the two diseases.

## Discussion

The present bioinformatic study applied immunocorrelation analysis to identify immune-related genes, cells and pathways that might serve as key linkage mechanisms between AD and PD. We found that innate immune cells M2 macrophages and NKT are highly expressed in both AD and PD. M2 macrophages are primarily involved in the Th2 immune response. Th2 cells produce cytokines that promote the humoral immune response, including IL-4, IL-5, IL-6, IL-10 and IL-13 (Sun et al., 2021). NKT cells mediate proinflammatory and immunomodulatory effects, which range from B-cell regulation, production of specific antibodies, suppression of autoimmunity to cytokine production, dendritic cell crosstalk, and T/B cell interactions (Seidel et al., 2020a). Infiltration of the brain by peripheral NK cells with altered cytotoxic properties has been documented as a contributory mechanism to neuroinflammation in AD but the specific roles of infiltrating NKT cells, which share phenotypic and functional properties are less well understood in AD (Busse et al., 2021; Lu et al., 2021). In PD, NKT cells are known to be activated by several Gram-negative periodontal pathogens can play proinflammatory roles (Seidel et al., 2020b). Of note, gene expression based immune cell infiltration analysis may include the extrapolation of certain genes that may also be expressed in non-immune cell lineages under conditions such as stress or inflammation, and this could account for the prediction of adipocytes and hepatocyte expression, which is unsupported by experimental evidence. For instance, ICAM-1, expressed on immune cell lineages, is overexpressed on adipocytes and hepatocytes (Farhood et al., 1995; Singh et al., 2023).

Among the adaptive immune cells, B-cells, CD4^+^ memory T-cells and CD8^+^ naive T-cells were found highly expressed in all 4 clusters of AD and PD. B cells might exert protective functions in periodontitis. B-cell-deficient mice show alveolar bone loss without bacterial infection, while clinical evidence shows that B cells and plasma cells, along with osteoclastogenic factors, are involved in alveolar bone destruction in periodontitis (Zouali, 2017). AD is associated with B cell accumulation in brain tissue which can produce IgG to induce microglial activation (Park et al., 2022). Experimental evidence supports the notion of infectious disease driven microglial activation in AD (Hao et al., 2022) along with peripheral leukocyte infiltration of brain tissue secondary to persistent systemic inflammation (Lu et al., 2021), as seen in periodontitis, in particular NK cell infiltration (Le Page et al., 2018), and our findings were largely consistent. High levels of NKT cell-related immune genes were also implicated in AD in our results, and aberrant NKT cell homeostasis has been reported in AD (Sh et al., 2021). The CD8^+^ naïve T cell subset was also overrepresented in AD samples, consistent with experimental evidence demonstrating CD8^+^ T cell infiltration of AD-affected brain parenchyma which have been associated with upregulated IFN-β signalling and infection (Altendorfer et al., 2022). Eosiniophils were also markedly overrepresented, and eosinophilic inclusions are well documented in Alzheimer’s neurofibrillary tangles (Qian et al., 2022). Eosinophilic signatures have also been inversely correlated with AD stage. The M2 macrophage signature noted in AD samples is corroborated by peripheral macrophage infiltration in experimental models of AD (Rentsendorj et al., 2018) and the M2 phenotype has been correlated with AD but not experimentally validated (Lin C. et al., 2022). M1/M2 phenotype switch is also a key feature of microglial changes in AD, and while *P. gingivalis* infection is associated with M1 type microglial switch, the stage of AD is an important determinant of M1/M2 microglial balance (Lin J. et al., 2022). Ageing and senescence are associated with deregulation of immune responses, and higher risk of both AD and PD. Furthermore, gender-based differences in AD pathology are recognised. A limitation of this investigation is that the datasets were not matched for age, gender, and disease stage which may induce confounding and should be addressed in future clinical investigations. Cluster analysis revealed innate immunity associated genes were comparatively highly expressed in cluster4 samples of AD and PD, and whether these samples represent a distinct phenotype, a later stage of disease progression, or represent more advanced age, begets further questions which should be dissected in future longitudinal studies to fully understand the PD-AD link.

Using a data mining approach with a series of reductive analyses, we obtained a 3 gene set, DUSP14, F13A1 and SELE, as key crosstalk genes linking PD and AD, which was largely supported by experimental and clinical data. The mechanistic role of DUSP14 in mediating AD and PD is not investigated but several DUSP genes are shown to be deregulated during AD pathogenesis (An et al., 2021). Targeting DUSP 14 can counter NLRP2 inflammasome mediated immune-inflammatory pathways and has shown positive effects in ameliorating neuroinflammtion and cognitive dysfunction (Que et al., 2020). There are few reports regarding the roles of DUSP14 in the literature, mainly focusing on pathways related to T cells. DUSP14 can downregulate T-cell receptor signalling by inhibiting TGF-β-activated kinase 1-binding protein 1 (TAB1) activation (Yang et al., 2014). DUSP14 is a mitogen-activated protein kinase phosphatase that plays a critical role in the regulation of T cell activity. TRAF2 mediated Lys63-linked ubiquitination of DUSP14 leads to DUSP14 activation in T cells (Yang et al., 2016). DUSP14 directly interacts with TGF-beta-activated kinase 1 (TAK1)-binding protein 1 (TAB1) and dephosphorylated TAB1 at Ser(438), leading to TAB1-TAK1 complex inactivation in T cells and can downregulate T-cell receptor (TCR) signalling by inhibiting TAB1 activation (Yang et al., 2014). Activated DUSP14 also directly dephosphorylates extracellular signal-regulated kinases (ERK) and attenuates the ERK signalling pathway. TRAF2-mediated ubiquitination of Lys63-linked DUSP14 also enhances its phosphatase activity (Chen et al., 2019). Protein arginine methyltransferase (PRMT)5-mediated arginine methylation may sequentially stimulate TRAF2-mediated DUSP14 ubiquitination and phosphatase activity, leading to inhibition of TCR signalling (Yang et al., 2018). Therefore, enhancement/activation of DUSP14 or DUSP14 upstream molecules is a potential modality for the attenuation of autoimmune diseases such as systemic lupus erythematosus (SLE) (Chuang and Tan, 2019).

F13A1 is involved in clot stabilization and implicated in a number of immunoinflammatory diseases (Dull et al., 2021). The role of F13A1 has been investigated in AD. F13A1 subunit was detected by immunohistochemistry in a subset of AD reactive microglia, while F13A1 Val34Leu gene polymorphism is associated with sporadic AD where homozygous LL genotype shows about a fourfold higher risk of developing AD compared to the homozygous VV genotype (Gerardino et al., 2006). F13A1 may also influence the maintenance of neural connections (Festoff et al., 2001). The F13A1 204Phe allele is strongly associated with ischemic stroke in young women and the homozygous genotype (Phe/Phe) are associated with manyfold higher stroke risk than heterozygous (Tyr/Phe) genotype (Pruissen et al., 2008). Functionally, a pro-angiogenic function of F13A1 is affected by the interaction between vascular endothelial growth factor receptor 2 (VEGFR2) and integrin αvβ3 on the cell membrane, which facilitates important steps in granulation tissue formation at wound sites. F13A1 deficiency can thus present as intracranial haemorrhage, delayed bleeding or chronic wounding of the skin and impaired mucosal healing. F13A1 thus functions to link primary hemostasis, coagulation, and definitive tissue healing. Another important recently identified function of F13A1 is its ability to control cellular infiltration by binding to specific macromolecules, thereby limiting bacterial spread at the wound site and promoting host cell migration and survival (Gemmati et al., 2016). In the brain, F13A1 expression has been detected by immunohistochemistry in reactive microglia during glioma formation, which is a distinctive feature of AD pathogenesis (Gerardino et al., 2006). F13A1 levels are gradually elevated from controls to mild cognitive impairment (MCI) and AD. More importantly, F13A1 in the serum proteome can serve as a potential non-invasive early diagnostic marker of MCI and AD (Kang et al., 2016). Of note, PD pathogens can induce the upregulation of the coagulation cascade-related genes in endothelial cells (Salmina et al., 2010) and may contribute the endothelial dysfunction inherent to AD pathogenesis (Hossain et al., 2020).

SELE encodes for E-selectin and is involved in Leukocyte/endothelial cell adhesion, and its expression is reported to increase 4-fold in *Treponema* denticola oral infections (Chukkapalli et al., 2014), a subgingival oral spirochete species which is a key periodontal pathogen (Zeng et al., 2021). Its role in several age-associated conditions such as age-related macular degeneration (Mullins et al., 2011) and other conditions. SELE has been found to be related with peripheral arterial occlusive disease (Shaker et al., 2010). The serum level of SELE has been found significantly elevated in systemic sclerosis with early onset disease (Hasegawa et al., 2014). Regarding periodontitis, the Ser128Arg polymorphism is associated with periodontitis (Houshmand et al., 2009). SELE expression is also found positively correlated with the duration of Sjogren’s syndrome, characterised by dysregulation of circulating immune cells, T cells and antigen presenting cells and vascular endothelial extravasation (Turkcapar et al., 2005; Blochowiak et al., 2017). In an animal model of AD, SELE expression was found significantly elevated, indicating its role in AD development (Wang et al., 2020). The cell-surface glycoprotein E selectin plays an important role in immune adhesion (McEver, 2015). It is also associated with the accumulation of white blood cells at sites of inflammation by mediating cell adhesion to the intima of blood vessels. As a clinical diagnosis, AD shows variable pathology. Clinically, E-selectin has been found significantly raised in the cerebrospinal fluid (CSF) of AD patients without typical signature biomarker profiles, suggesting it may specifically mark the vascular mechanisms underlying AD pathology (Li et al., 2015). The SELE Ser128Arg gene polymorphism has also been associated with AD (Horstmann et al., 2010; Ribizzi et al., 2010; Flex et al., 2014) and SELE polymorphisms are also associated with Lewy body dementias (Rajkumar et al., 2020). The findings of the functional correlation analysis indicate an interaction between these candidate biomarker genes with key pathways intersecting adaptive immune responses, TNF alpha mediated inflammation, and endothelial dysfunction, supporting PD infection-mediated systemic immune dysregulation at the core of the AD-PD link.

Overall, the findings of this bioinformatic study were supported by exisiting experimental evidence addressing PD and AD but the roles of the discovered biomarkers DUSP14, F13A1 and SELE in mediating the link between the two diseases has not been addressed. A major limitation of the present study is the lack of validation experiments using cell, animal or clinical data. Therefore, the present data must be considered as a theoretical premise for further investigation that explores the validity of these biomarkers in large-scale clinical trials and their mechanistic roles in experimental or translational research focused on immune mechanisms implicated in AD and PD linkage.

## Conclusion

Bioinformatic analysis integrating experimental transcriptomic data from Alzheimer’s disease and periodontitis revealed the most robust potentially shared molecular linkages. Three biomarker crosstalk genes; DUSP14, F13A1 and SELE were identified as the most robust signature. Macrophages M2 and NKT among innate immune cells, and B-cells, CD4^+^ memory T-cells and CD8^+^ naive T-cells among adaptive immune cells emerged as top immune cells linking PD and AD. These findings warrant future research in experimental and clinical studies.

## Data Availability

The datasets presented in this study can be found in online repositories. The names of the repository/repositories and accession number(s) can be found in the article/Supplementary Material.
